# Pronounced antiseizure activity of the subtype‐selective GABA_A_ positive allosteric modulator darigabat in a mouse model of drug‐resistant focal epilepsy

**DOI:** 10.1111/cns.13927

**Published:** 2022-08-14

**Authors:** Rachel Gurrell, Philip Iredale, Alexis Evrard, Venceslas Duveau, Céline Ruggiero, Corinne Roucard

**Affiliations:** ^1^ Cerevel Therapeutics Cambridge Massachusetts USA; ^2^ SynapCell Saint Ismier France

**Keywords:** CVL‐865, darigabat, drug‐resistant epilepsy, focal, gaba, GABA, MTLE, seizure (total ≥5, ≤8)

## Abstract

**Aim:**

Darigabat is an α2/3/5 subunit‐selective positive allosteric modulator of GABA_A_ receptors that has demonstrated broad‐spectrum activity in several preclinical models of epilepsy as well as in a clinical photoepilepsy trial. The objective here was to assess the acute antiseizure effect of darigabat in the mesial temporal lobe epilepsy (MTLE) mouse model of drug‐resistant focal seizures.

**Methods:**

The MTLE model is generated by single unilateral intrahippocampal injection of low dose (1 nmole) kainic acid in adult mice, and subsequent epileptiform activity is recorded following implantation of a bipolar electrode under general anesthesia. After a period of epileptogenesis (~4 weeks), spontaneous and recurrent hippocampal paroxysmal discharges (HPD; focal seizures) are recorded using intracerebral electroencephalography. The number and cumulated duration of HPDs were recorded following administration of vehicle (PO), darigabat (0.3–10 mg kg^−1^, PO), and positive control diazepam (2 mg kg^−1^, IP).

**RESULTS:**

Darigabat dose‐dependently reduced the expression of HPDs, demonstrating comparable efficacy profile to diazepam at doses of 3 and 10 mg kg^−1^.

**CONCLUSIONS:**

Darigabat exhibited a robust efficacy profile in the MTLE model, a preclinical model of drug‐resistant focal epilepsy. A Phase II proof‐of‐concept placebo‐controlled, adjunctive‐therapy trial (NCT04244175) is ongoing to evaluate efficacy and safety of darigabat in patients with drug‐resistant focal seizures.

## Graphical abstract

The antiseizure effect of darigabat, an α2/3/5 subunit‐selective GABA_A_ receptor positive allosteric modulator, was examined in the mesial temporal lobe epilepsy mouse model of drug‐resistant focal seizures. Darigabat exhibited a robust dose‐dependent reduction in focal seizures (hippocampal paroxysmal discharges) in the model, indicating that selective modulation of GABA_A_ receptors may have a clinical utility in patients with drug‐resistant focal seizures.

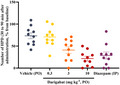

## 1 INTRODUCTION

Despite the development of more than a dozen new antiseizure medications (ASMs) in the last 30 years, roughly a third of people with epilepsy continue to experience pharmacoresistance, and thus, drugs that are effective in controlling treatment‐resistant seizures continue to represent a significant unmet medical need. Historically, preclinical screening of novel compounds with potential antiseizure activity has been dominated by acute models such as the maximal electroshock seizure (MES) and pentylenetetrazol (PTZ) models that induce seizures in healthy, neurologically intact rodents and have been in use for >80 years. Without question, these and similar preclinical models have played an important role in the identification of multiple ASMs that have benefitted millions of people with epilepsy across the world. However, more recently, sophisticated chronic disease‐relevant nonclinical models of pharmacoresistant seizures have been incorporated into early drug discovery programs to help identify promising new compounds with the potential to control drug‐resistant seizures.^1^, ^2^ One such model that has key features to address this translational gap is the mesial temporal lobe epilepsy (MTLE) mouse model.

This model is generated by a single unilateral injection of a small dose of kainic acid (1 nmole) in the dorsal hippocampus of mice to reproduce many of the morphological and electrophysiological features of clinical MTLE (reviewed in^3^). These translationally relevant features include cell losses in CA3 and CA1, dispersion of the granule cells layer in the dentate gyrus and sprouting of their terminals, and spontaneous and recurrent hippocampal paroxysmal discharges (HPDs). Approximately 3 weeks after kainic acid injection, the HPDs, which last approximately 15–20 s, can be recorded using electroencephalography (EEG). These focal seizures occur frequently (average 40/h) and remain stable during the rest of the animal's life.^3^ Importantly, the MTLE mouse model is resistant to most classical ASMs, with acute administration of valproate, carbamazepine, and lamotrigine only suppressing the hippocampal discharges at high doses that are also associated with side effects.^3^, ^4^ Conversely, more recently developed ASMs such as pregabalin and levetiracetam, and a broad class of drugs that collectively facilitate GABAergic transmission, including phenobarbital, diazepam, tiagabine, and vigabatrin, suppressed HPDs in a dose‐dependent manner at doses devoid of obvious behavioral effects.^3^ If applying the operational definition of ASM resistance in patients with epilepsy^5^ in the context of animal models, it would be described as recurrent seizures not responding to, or with poor treatment response to, at least two ASMs at tolerated doses.^6^ Given this mouse model presents with behavioral, electrophysiological, and pharmacological features of drug‐resistant focal epilepsy, it is a favorable model to enable identification of potential novel ASMs for the treatment of drug‐resistant focal seizures.

GABA_A_ receptors are heteropentameric ligand‐gated ion channels assembled from 19 members of the GABA_A_ receptor family (α1‐6, β1‐3, γ1‐3, δ, ε, θ, π, and ρ1‐3) with the most abundant subtypes comprising α, β, and γ subunits in a 2:2:1 stoichiometry.^7^ Activation of GABA_A_ receptors by its natural ligand GABA leads to increased membrane chloride conductance through the integrated chloride channel, which (in mature neurons) results in membrane hyperpolarization, a subsequent decrease in the probability of further action potentials occurring and a dampening down of excitability. Benzodiazepines (BZDs), non‐selective positive allosteric modulators (PAMs) of GABA_A_ receptors, bind to an allosteric site on GABA_A_ receptors and enhance chloride conductance by increasing the affinity of the receptor for its natural ligand GABA. BZDs have been used clinically since the 1960s, and although they are effective antiseizure medications and anxiolytics, their use is severely limited by the potential for side effects, including sedation, addiction, and loss of efficacy, particularly in the treatment of seizures. Sophisticated molecular studies in which specific alpha subunits have been rendered unresponsive to diazepam^8^ have attributed the sedative effects of BZDs to α1 activity,^9^ antiseizure activity to α1 and α2 subunits (α1/2^10^;), anxiolytic effects to α2/3,^11^, ^12^, ^13^ addictive properties to α1/2 subtypes,^14^, ^15^ and development of efficacy tolerance in epilepsy populations to full agonist‐like activity at the α1 subunit.^16^, ^17^ As such, there has been a concerted effort to identify the next generation of GABA_A_ receptor α2/3 subtype‐selective PAMs, with negligible potentiation of GABA via the α1 subunit and an optimized level of GABA potentiation (e.g., “partial modulation” vs full agonist‐like activity) in a bid to chronically treat seizures and anxiety with an improved side‐effect profile versus a BZD.

Darigabat (formerly CVL‐865 and PF‐06372865) is a novel small molecule rationally designed with both functional selectivity in vitro and in vivo for receptors containing α2/3/5 subunits compared with those containing the α1 subunit and optimized lower functional GABA potentiation than a BZD.^18^, ^19^ It has demonstrated antiseizure activity in a broad spectrum of rodent models, including amygdala kindling, pentylenetetrazol (PTZ), and in the genetic absence epilepsy GAERS model.^19^, ^20^ Furthermore, darigabat has demonstrated robust clinical activity in a proof‐of‐principle study in patients with photosensitive epilepsy.^21^ Darigabat has been shown to be generally well‐tolerated in both healthy volunteers and patients in the clinic.^18^, ^22^, ^23^, ^24^ In this study, we evaluated for the first time whether selective enhancement of the inhibitory effect of GABA_A_ α2/3/5 receptors would suppress the aberrant overexcitation that underlies epileptiform activity in a mouse model of treatment‐resistant focal seizures.

## 2 MATERIALS AND METHODS

### 2.1 Animals

Adult male C57BL/6J mice aged 11 weeks were purchased from Janvier (Le‐Genest‐St‐Isle, France) and were allowed to acclimate for at least 1 week before experiments. Prior to surgery, animals were socially housed in cages with wood litter and ad libitum access to food and water. Cages were maintained under artificial lighting between 7:30 a.m. and 7:30 p.m. in a room with controlled ambient temperature (22 ± 2°C) and relative humidity.

All experiments were approved by the ethical committee of the High Technology Animal Platform, University Grenoble Alpes, and performed in accordance with the European Committee Council directive of September 22, 2010 (2010/63/EU). All efforts were made to minimize animal suffering and reduce the number of animals used.

### 2.2 Compounds and administration

Darigabat was provided by Cerevel Therapeutics (Cambridge, MA). Diazepam was purchased from Roche. Darigabat (0.3, 3, and 10 mg kg^−1^; PO) was dissolved in a solution containing 17% Kolliphor HS 15, 18% glycerol formal in water and diazepam (2 mg kg^−1^, IP) in saline immediately before administration to the animals (dose volume 10 ml/kg). The vehicle was a solution of 17% Kolliphor HS 15, 18% glycerol formal in water. Maximal plasma concentration and brain receptor occupancy of darigabat was anticipated to be at approximately 1 h after oral dosing. Differing route of administration was required for darigabat and diazepam to achieve targeted plasma exposure levels. The doses of darigabat were selected based on previously published data.^19^ 2 mg kg^−1^ diazepam was selected based on prior data indicating robust antiseizure activity at this dose devoid of side effects.^3^ Animals were randomized to treatment order using a Latin‐square cross‐over protocol and blinding to treatment conditions were maintained throughout data acquisition and analysis. A washout period of 3 to 4 days was allowed between drug administrations.

### 2.3 Surgery

Under general anesthesia (isoflurane in 2%–3% oxygen), mice were stereotaxically injected with kainic acid (KA) (1 nmole of KA dissolved in NaCl solution [0.9%]; Sigma‐Aldrich, Lyon, FR, USA) into the right dorsal hippocampus (AP = −2, ML = −1.5, DV = −2 mm from bregma) as previously described.^3^

After KA injection, mice were implanted with stainless steel bipolar electrode (PlasticOne) into the right dorsal hippocampus. The recording system was then fixed to the skull with dental cement.

### 2.4 EEG recording

Animals were used between 4 and 6 weeks after the intrahippocampal injection and electrode implantation.

On the day of the recording session, the selected animals were placed in a recording chamber and connected to their cable. A 1‐h habituation period was allowed before the EEG recording session. EEG recordings were performed on freely moving animals for 20 min pre‐administration (baseline period) and for 130 min post‐administration using SystemPlus Evolution (Micromed France, Mâcon, France / 512 Hz sampling rate, low pass filter).

**FIGURE 1:**
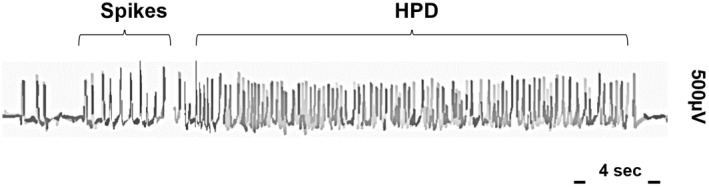
Example of a typical HPD in an EEG recording in a MTLE mouse selected in the study prior to compound administration. The HPD occurs spontaneously, with an obvious start and well‐defined stop over the basal EEG.

An example of a typical hippocampal paroxysmal discharge obtained by EEG in an MTLE mouse selected for the study is shown in Figure 1. The HPDs observed under control conditions occurred spontaneously, with a clearly defined beginning and end.

### 2.5 Data analysis

EEG recordings were analyzed offline and quantified blindly by an expert on SynapCell's proprietary platform to identify HPDs.^3^

HPDs were analyzed during a 20‐min baseline period (immediately before compound administration) and for a period of 120 min between 10 and 130 min after compound administration. The first 10 min after administration was not included in the analysis due to the potential impact of dosing on the HPDs. For each animal and administration, data were computed for the number of HPDs per 20‐min period.

### 2.6 Statistical analysis

Data were expressed as mean ± SEM. Statistical analysis was performed using Prism 9.1.0 (Graphpad). First, the normality of the data was tested and confirmed using the Shapiro–Wilk test. The data from the time course were analyzed using the Friedman test (non‐parametric) followed by paired comparisons vs vehicle using the Dunn's test. The total number and cumulated duration of HPDs during the 30‐90‐min time period were compared using a one‐way ANOVA for repeated measures, followed by paired comparisons vs baseline periods, vs vehicle, and vs diazepam using the Bonferroni's t‐test. The significance level was set at *p* < 0.05.

### 2.7 Plasma sample collection and bioanalysis

To confirm that the plasma exposures achieved were as previously expected, blood was collected from each animal at the end of the last EEG recording (~2.5 h post‐administration). To collect blood, the animals were deeply anesthetized with isoflurane (2%–3% in oxygen) and a terminal blood sample was collected by in K2/EDTA tubes, and immediately stored on ice. Within 10 min after collection, blood samples were centrifuged at 4°C and 3000 g for 10 min, and the plasma was collected and divided in 2 plastic tubes. All plasma samples were stored at −80°C until analysis.

Plasma samples were analyzed by protein precipitation with volumes of internal standard containing acetonitrile (5:1 ratio with sample), followed by mixing and centrifugation to pellet the protein. Supernatant was then mixed (1:1) with water prior to analysis by LC–MS/MS via a multiple reaction monitoring transition method for darigabat: 441.4 > 348.2. Limits of quantification of 0.5 ng ml^−1^ were achieved.

## 3 RESULTS

All the MTLE mice enrolled in this study demonstrated prototypical HPDs prior to compound administration (Figure 1) and exhibited at least 20 HPDs per hour during a preliminary EEG recording. The selected mice were then included in a cross‐over protocol with the aim of evaluating the acute effect of darigabat. A total of 10 MTLE mice completed the cross‐over protocol.

### 3.1 Effect of darigabat on the number of HPDs

**FIGURE 2:**
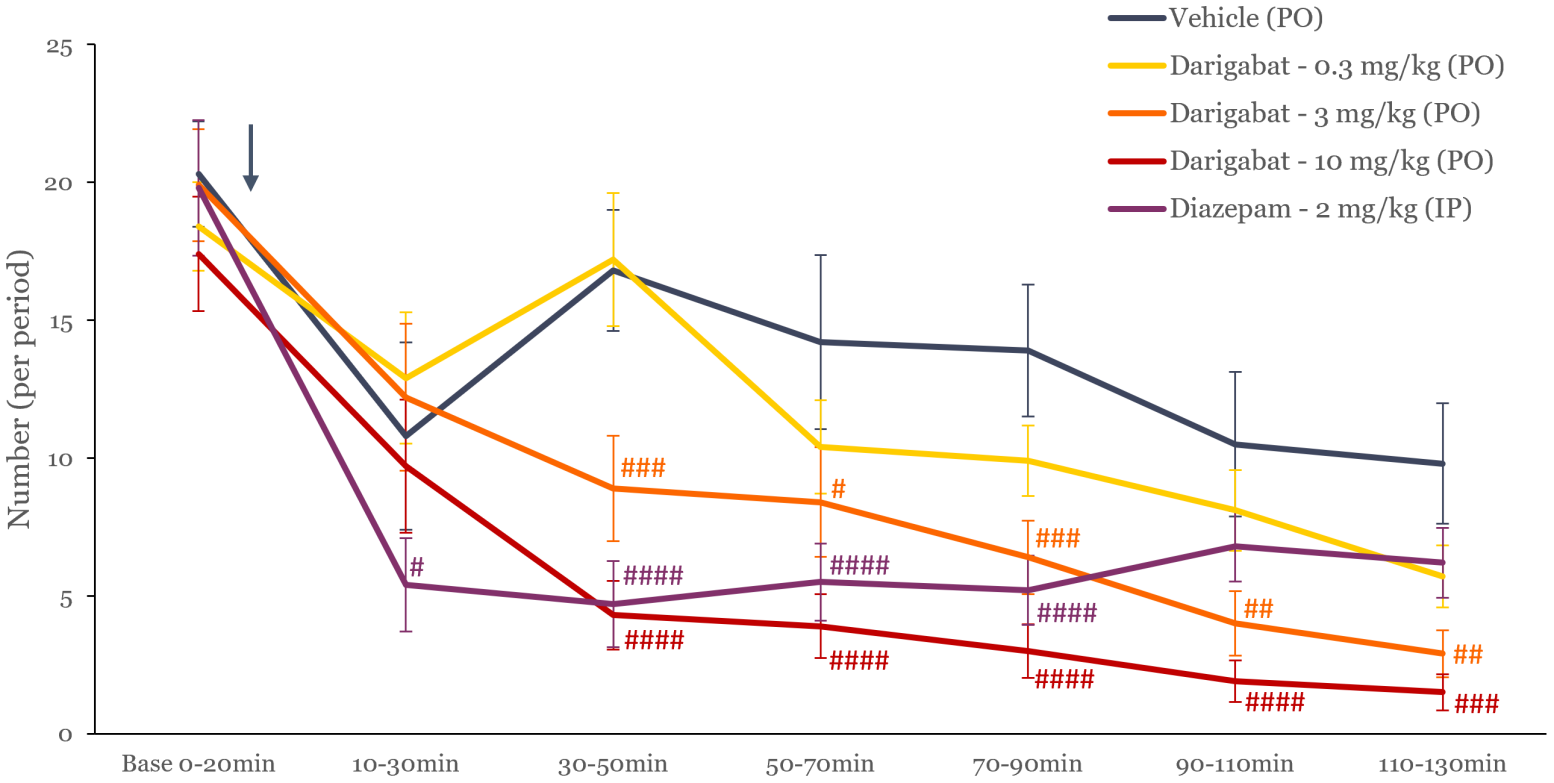
Time course of the effect of compound over time on number of HPDs. Time course of the effect of vehicle, darigabat (0.3, 3, 10 mg kg^−1^, PO), and diazepam (2 mg kg^−1^, IP) on the number of hippocampal paroxysmal discharges (HPD; mean ± SEM, *n* = 10) during baseline and post‐administration periods. The arrow indicates the timing of compound administration. #, ##, ###, ####: *p* < 0.05, 0.01, 0.001, 0.0001, respectively, as compared to vehicle using a two‐way ANOVA.

The first parameter that was measured on EEG recordings was the effect of compound administration on the number of HPDs over time, Figure 2 and Table 1.

**TABLE 1:** Number of HPDs for all treatments tested over time

	Darigabat (PO)				Diazepam (IP)
	Vehicle	0.3 mg kg−1	3 mg kg−1	10 mg kg−1	2 mg kg−1
	Mean (SEM)	Mean (SEM)	Mean (SEM)	Mean (SEM)	Mean (SEM)
Pre‐dose Baseline 0–20 min	20.30 (1.91)	18.40 (1.61)	19.90 (2.03)	17.40 (2.08)	19.80 (2.47)
10–30 min	10.80 (3.40)	12.90 (2.39)	12.20 (2.67)	9.70 (2.42)	5.40 (1.69)
30–50 min	16.80 (2.19)	17.20 (2.41)	**8.90** (1.91)	**4.30** (1.24)	**4.70** (1.56)
50–70 min	14.20 (3.16)	10.40 (1.69)	**8.40** (1.98)	**3.90** (1.16)	**5.50** (1.39)
70–90 min	13.90 (2.39)	9.90 (1.29)	**6.40** (1.33)	**3.00** (0.97)	**5.20** (1.25)
90–110 min	10.50 (2.62)	8.10 (1.46)	**4.00** (1.17)	**1.90** (0.75)	6.80 (1.28)
110–130 min	9.80 (2.18)	5.70 (1.13)	**2.90** (0.85)	**1.50** (0.65)	6.20 (1.27)

*Note*: Data expressed as mean number ± SEM, *n* = 10 MTLE. Bold number indicates *p* < 0.05 as compared to vehicle.

Darigabat induced a dose‐dependent reduction in the number of HPDs for at least 130 min post‐administration. At the low dose (0.3 mg kg^−1^), a significant decrease versus baseline but not versus vehicle was observed, potentially due to a reduction in HPDs observed in the vehicle group over time. The intermediate dose of darigabat (3 mg kg^−1^) showed a significant reduction in the number of HPDs between 70 min and for the rest of the duration of the recording (130 min) (P at least <0.05 for all the time points). The highest dose of darigabat (10 mg kg ^−1^) displayed a longer effect starting 30 min after administration and was observed until the end of the recording (130 min post‐administration). The positive control diazepam induced a significant reduction in the number of HPDs, which was sustained for a duration of approximately 90 min after dosing in line with the expected pharmacokinetic profile. A similar effect profile for both darigabat and diazepam was observed in the duration of HPDs (Table 2). Although there was some instability observed in the vehicle group at the later time points of the study, this did not confound the ability to discriminate between vehicle and treatment groups. There were no observable side effects induced by any of the treatments administered.

**TABLE 2:** Cumulative duration of HPDs for all treatments tested over time

	Darigabat (PO)				Diazepam (IP)
	Vehicle	0.3 mg kg−1	3 mg kg−1	10 mg kg−1	2 mg kg−1
	Mean (SEM)	Mean (SEM)	Mean (SEM)	Mean (SEM)	Mean (SEM)
Pre‐dose Baseline 0–20 min	286.9 (31.3)	222.8 (24.7)	271.5 (29.3)	259.0 (34.0)	256.1 (35.2)
10–30 min	102.8 (34.8)	122.1 (26.5)	132.1 (28.0)	106.4 (21.3)	70.6 (21.5)
30–50 min	193.9 (32.7)	200.2 (25.0)	**99.9** (20.6)	**59.3** (16.8)	**53.0** (18.4)
50–70 min	154.8 (35.8)	148.4 (32.6)	93.6 (16.1)	**68.7** (27.7)	**85.9** (22.4)
70–90 min	169.4 (31.9)	119.5 (23.6)	**73.1** (15.5)	**48.0** (17.1)	**80.0** (18.8)
90–110 min	150.8 (35.6)	111.7 (26.5)	**56.1** (17.2)	**33.6** (15.0)	124.0 (29.6)
110–130 min	123.2 (30.5)	88.2 (24.8)	**43.5** (17.3)	**16.1** (6.3)	105.0 (24.2)

*Note*: Data expressed as mean duration in seconds ± SEM, *n* = 10 MTLE. Bold number indicates *p* < 0.05 as compared to vehicle.

### 3.2 Dose–response effect of darigabat

**FIGURE 3:**
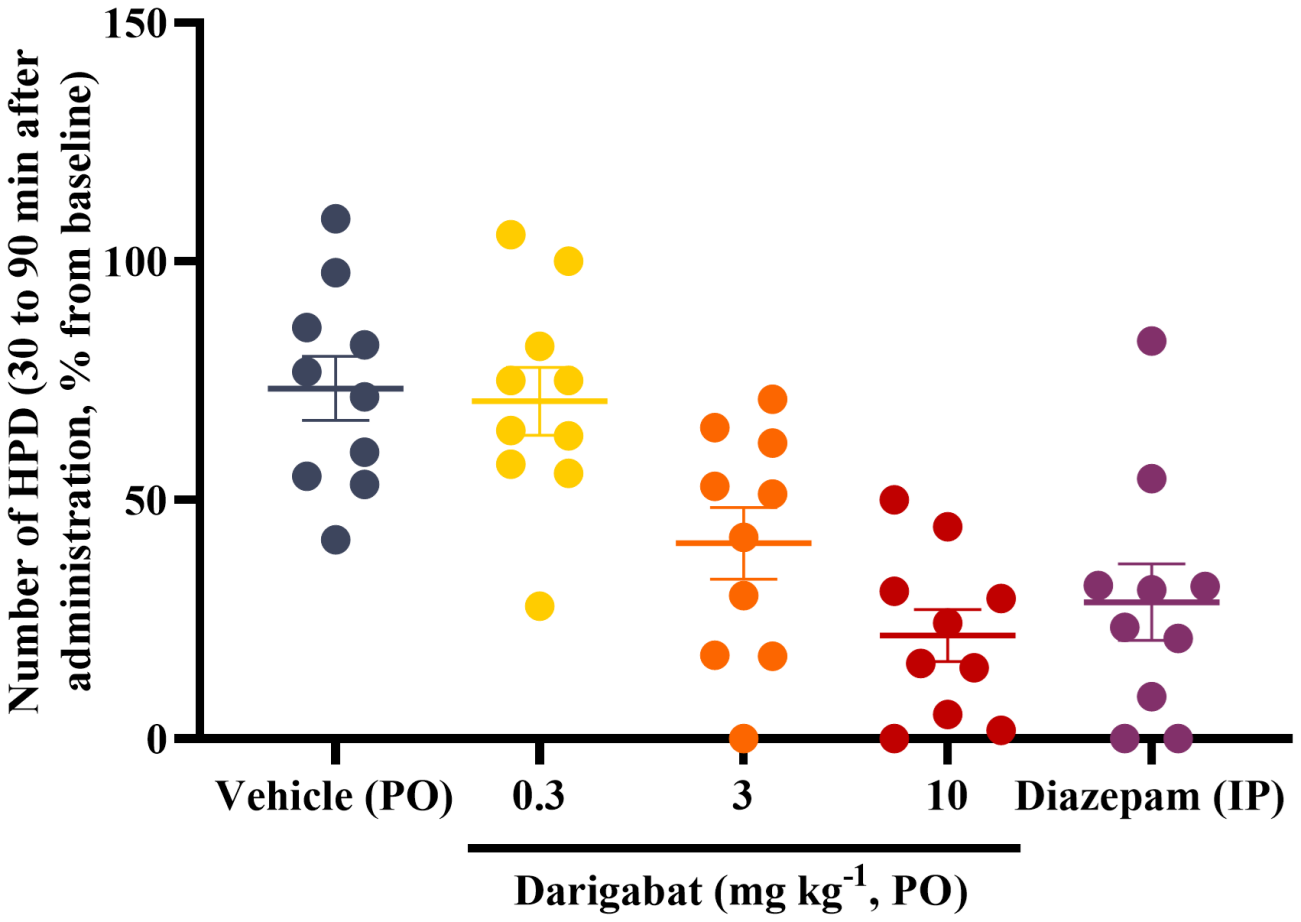
Effect of darigabat on number of HPDs. Dot plot (individual rodent) of hippocampal paroxysmal discharges (HPDs) during the 30‐ to 90‐min period after administration was measured and expressed as a % of the baseline HPD frequency (mean ± SEM, *n* = 10). #, ##, ###: *p* < 0.05, 0.01, and 0.001, respectively, as compared to vehicle using a one‐way ANOVA. Diazepam was administered 2 mg kg^−1^, IP.

Similarly, darigabat dose‐dependently reduced the frequency of HPDs (Table 1; Figure 3 for cumulative frequency of HPD over 1 h) and duration of HPDs (Table 2). Both 3 and 10 mg kg^−1^ darigabat induced a significant reduction in number and cumulative frequency of HPDs at peak time (between 30 and 90 min) compared with baseline and vehicle, and similar in magnitude to diazepam.

### 3.3 Plasma exposure of darigabat

At 10 mg kg^−1^, the means plasma exposure at approximately 2.5‐h post‐administration was 1793 ng ml^−1^ (SEM 147), which was within 2‐fold of that previously reported.^19^ The corresponding plasma exposures at 0.3 and 1 mg kg^−1^ were 51 and 586 ng ml^−1^, respectively, and were within the expected range.

## 4 DISCUSSION

To our knowledge, this is the first preclinical study of an α2/3/5‐subtype‐selective GABA_A_ receptor PAM in a rodent model of treatment‐resistant focal epilepsy. A robust efficacy profile was observed with darigabat, which was similar in magnitude to that obtained with diazepam, however with a longer‐lasting effect. Furthermore, the effect was observed using only a small number of animals without the induction of any observable side effects.

Darigabat dose‐dependently decreased the number of HPDs when administered to MTLE mice. There was no significant effect on the expression of HPDs compared with vehicle within the timeframe that was examined at the lowest dose of 0.3 mg kg^−1^, which was expected to achieve total receptor occupancy at GABA_A_ receptors in brain of approximately 48% based on data previously reported in mouse.^19^ However, at the two highest doses tested, 3 and 10 mg kg^−1^, that are estimated to achieve approximately 75 and 95 % receptor occupancy, respectively, the HPD inhibition was significant at the early time points, and the reduction was complete and persistent from 30 to 130 min after administration (the duration of the recording). Darigabat's effect was sustained at the 130 min time point unlike that of diazepam that appeared to diminish, suggesting an overall longer‐lasting response and in line with the pharmacokinetic properties reported for both drugs.^18^, ^25^

The high levels of darigabat receptor occupancy required to observe efficacy is in contrast with the low (estimated <10%) levels required for the non‐selective BZD positive control diazepam to produce similar levels of efficacy. This likely reflects the differential pharmacology between darigabat and BZDs, in terms of both the GABA_A_ receptor α subunit selectivity profile, but also the comparative levels of functional potentiation of GABA. Darigabat was designed to lack α1 activity to minimize sedation and other negative attributes associated with BZDs while retaining the robust antiseizure and anxiolytic activity they are associated with via potentiation at α2‐ and α2/3‐subunit containing GABA_A_ receptors, respectively. However, darigabat also has comparatively lower functional activity at α2/3/5‐containing receptors compared with BZDs.^18^ This is an important design attribute that is hypothesized to be associated with a lower propensity for efficacy tolerance,^17^ an issue that prevents the chronic usage of BZDs in many epilepsy patients. Furthermore, there is evidence that PAMs with lower functional activity need to occupy a greater proportion of the receptors to produce the same behavioral effect as a PAM with higher functional activity in nonclinical models of anxiety^26^ and pain.^27^ The preclinical epilepsy model data with darigabat are aligned with this, as evidenced by approximately >50% receptor occupancy being required to observed efficacy in rodent amygdala kindling, PTZ, and genetic absence epilepsy GAERS models^19^, ^28^ and the current data reported here in the MTLE mouse model. This suggests that there is potentially a receptor occupancy threshold under which GABA_A_ receptor PAMs with lower functional activity than BZDs are not effective.

A proof‐of‐principle clinical trial in the photosensitivity model has been conducted with darigabat.^21^ Briefly, the photosensitivity model enrolls participants who have reproducible generalized epileptiform discharges on EEG provoked by photic stimulation with flashing lights. Given that pharmacologic response in this model has been demonstrated to substantially increase the likelihood that efficacy will be seen in the clinical epilepsy population for a range of antiseizure mechanisms, single‐dose trials have been utilized in early clinical development as a reliable indicator of antiseizure effect.^29^, ^30^ Single doses of 17.5 mg darigabat (~60% RO) and 52.5 mg darigabat (~80% RO) were associated with a marked and statistically significant reduction in photosensitivity compared with placebo, that was similar in degree to the positive control lorazepam, and full abolition of the photosensitivity response in 6 out of 7 patients. Lower doses were not characterized in that clinical trial; however, it may be that the requirement for high receptor occupancy in nonclinical epilepsy models translates to significant antiseizure activity only being obtained in humans at very high receptor occupancy.

It has previously been reported that ASMs that facilitate GABAergic transmission have demonstrated particularly robust efficacy profile in the MTLE model,^3^ and these data with darigabat are supportive of that observation. First (phenobarbital), second (BZDs), and third (tiagabine and vigabatrin) generation GABAergic ASMs non‐selectively enhance GABA via positive allosteric modulation of GABA at specific binding sites on GABA_A_ receptors (barbiturates and BZDs), or by increasing the availability of GABA at the synapse by preventing its reuptake (tiagabine) or preventing its breakdown (vigabatrin). This is translationally relevant in that, for example, both tiagabine and vigabatrin have been approved for the adjunctive treatment of focal onset seizures. The next generation of GABAergic modulators have had varying success in the clinic. For example, padsevonil, that is reported to have a dual action of high‐affinity binding at SV2 synaptic vesicles and potentiation of GABA by non‐selective binding to BZD‐sensitive GABA_A_ receptors, recently failed to meet its primary endpoint in a Phase 2b trial in patients with refractory focal epilepsy despite demonstrating efficacy in the MTLE model.^31^, ^32^ The reasons for this failure of translation could include the very low levels (< 10%) of GABA_A_ receptor occupancy expected for padsevonil given that it possesses much lower levels of intrinsic functional activity than a BZD.^31^ Furthermore, the clinical trial enrolled patients that had failed at least 4 ASMs previously (which could represent a highly refractory population) and permitted the inclusion of both patients that were administering SV2A modulators (brivaracetam and levetiracetam) as stable background ASMs, and those that were resistant to other SV2A modulators which may have confounded the ability of padsevonil to demonstrate efficacy in this population. By contrast, cenobamate, that also has a dual mechanism of GABA enhancement (via a distinct mechanism from BZDs) and preferential inhibition of persistent sodium channel, has exhibited robust efficacy in several randomized controlled trials in patients with focal epilepsy.^32^ Cenobamate has not been profiled in the MTLE mouse model and that would be of interest given its robust reduction in seizures in drug‐resistant epilepsy patients and mechanism of action. Given that the current study is acute and in rodents only, it is not yet confirmed that the translation of efficacy will hold for darigabat until clinical data from a clinical trial in patients with drug‐resistant focal epilepsy are available. Furthermore, it would be of interest to examine the effect of chronic administration of darigabat in the mouse MTLE model.

Millions of patients worldwide have benefited from successful translation of antiseizure activity from a battery of nonclinical epilepsy models to efficacy in the clinic. However, given that approximately 30% of patients with epilepsy continue to have suboptimal therapeutic benefit with current ASMs, there needs to be continued evolution of drug development, ideally with the use of models that facilitate the identification of novel ASMs for the symptomatic treatment of patients with refractory seizures. Due to the advantages of the MTLE mouse model in terms of both the morphological, electrophysiological, and drug‐resistant pharmacology profile, this model is currently being utilized as a mouse model of therapy‐resistant focal epilepsy by the Epilepsy Therapy Screening Program administered by NIH/NINDS,^33^ which was established to facilitate the discovery of new therapeutic agents addressing the unmet medical needs in epilepsy.

There is optimism that darigabat will show potential in patients with drug‐resistant focal epilepsy based on positive data in the MTLE mouse model described here and the robust activity observed in the photosensitivity model with darigabat.^21^ As such, a Phase II proof‐of‐concept placebo‐controlled, adjunctive‐therapy trial (CVL‐865‐SZ‐001, NCT04244175) has been initiated and is evaluating the efficacy and safety of darigabat at doses achieving approximately 60 and 80% receptor occupancy in patients with drug‐resistant focal seizures. An open‐label extension study (CVL‐865‐SZ‐002, NCT04686786) will evaluate long‐term safety as an extension to the Phase 2 proof‐of‐concept trial.

## AUTHOR CONTRIBUTIONS

RG, PI, AE, and VD designed the protocol. CRu and BMN executed the protocol. AE, CRu, and CD analyzed the data. RG drafted the manuscript for review by PI, VD, CR, and AE.

## CONFLICT OF INTEREST

RG and PI are or were employees of Cerevel Therapeutics at the time of this research and may own stock and/or stock options in the company.

## Data Availability

Data available on request from the authors
